# Pioneering Role of T.C. Merigan in the Treatment of Various Virus Infections

**DOI:** 10.3390/molecules31010025

**Published:** 2025-12-22

**Authors:** Erik De Clercq

**Affiliations:** Rega Institute for Medical Research, Department of Microbiology, Immunology and Transplantation, KU Leuven, B-3000 Leuven, Belgium; erik.declercq@kuleuven.be

**Keywords:** interferon, pyran copolymer, poly r($\bar{s}$A-$\bar{s}$U), VZV, HSV, CMV, HBV, HIV

## Abstract

The research of Prof. Dr. Thomas C. Merigan has spanned almost half a century. It started in 1963 with his interest in interferon (*i*). He then identified pyran copolymer as a synthetic polyanionic inducer of interferon (*ii*), and thereafter thiophosphate-substituted polyribonucleotides, i.e., poly r($\bar{s}$A-$\bar{s}$U) (*iii*). He recognized the potential of interferon as a therapeutic agent for virus infections (*iv*), varicella-zoster virus (VZV) being the first case in point (*v*). His interest then shifted to the treatment of herpes virus [herpes simplex virus (HSV) and cytomegalovirus (CMV)] infections (*vi*) and hepatitis B virus (HBV) infections (*vii*), to end up with human immunodeficiency virus (HIV) infections (*viii*, *ix*, *x*). T.C. Merigan’s pioneering work on the treatment of so many pivotal virus infections deserves further in-depth clinical evaluation.

## 1. Introduction

**Figure 1:**
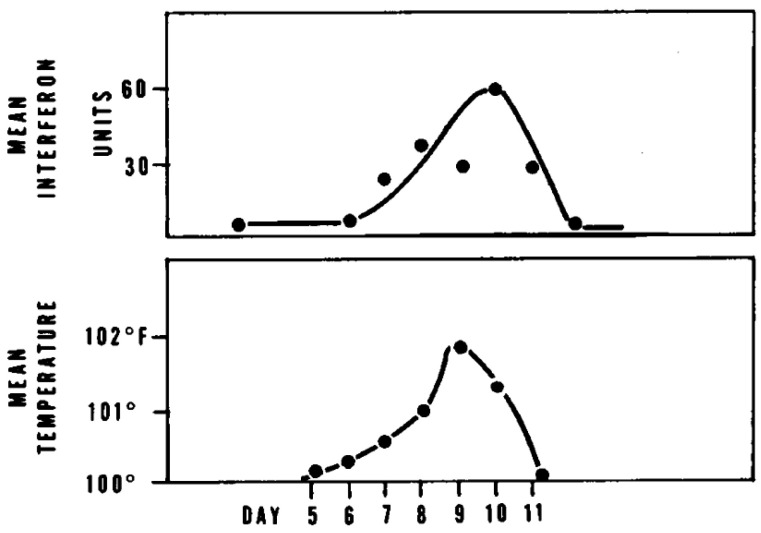
Correlation of serum interferon and fever responses after measles vaccination. Reproduced with permission from J. K. Petralli, T. C. Merigan and J. R. Wilbur, *The New England Journal of Medicine*; published by Massachusetts Medical Society, 1965 [2].

T.C. Merigan first reported that highly purified preparations of chick and mouse interferons had marked species specificity [1]. He concluded that interferon is a virus-induced product of the host genome [1]. Petralli et al. [2] then detected a viral inhibitory factor in the serum of 17 of 18 children undergoing primary measles vaccination; this viral inhibitory factor had the properties of human interferon [2]: its appearance closely correlated with the induction of fever (Figure 1). Its presence 6 to 11 days after vaccination was consistent with the hypothesis that it mediates the interference observed when vaccination with attenuated measles virus prevents the clinical manifestations of measles even if given 3 days after exposure [2]. The measles virus-stimulated circulating interferon was also associated with protection against vaccinia virus infection [3].

## 2. Pyran (Maleic Divinyl Ether) Copolymer

**Figure 2:**
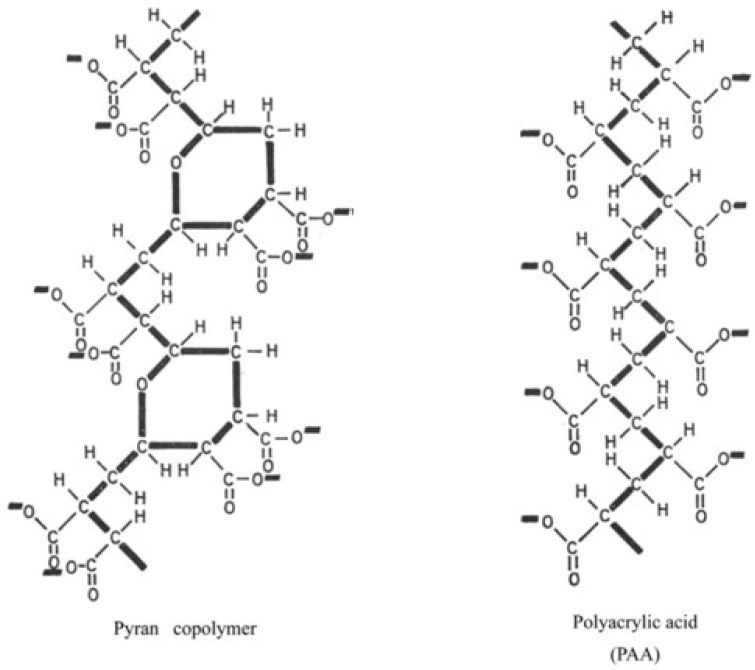
Molecular structure of pyran (maleic anhydride/divinyl ether) copolymer and polyacrylic acid (PAA). Original work from E. De Clercq.

While reviewing interferons of mice and men [4], Merigan referred to a synthetic polysaccharide of 17,000 molecular weight, found by Dr. William Regelson, to be capable of inducing circulating antiviral activity in the mouse. That this “synthetic anionic polymer of known composition” might act as an interferon inducer in humans was implied by T.C. Merigan [5]. That it actually did so was eventually demonstrated by Merigan and Regelson [6]. Protection against Mengo virus infection in the mouse was found with doses of pyran copolymer as low as 1 mg per kg of body weight [6]. This suggests that antiviral effects in man might be achieved at lower (and probably safer) dosage levels than those required to produce demonstrable circulating interferon [6]. The news that other synthetic anionic polymers, such as polyacrylic acid, were also capable of inducing interferon [7,8] must have reached T.C. Merigan by 1968, so that he and Finkelstein referred to polyacrylic acid in their paper [9]. I included the chemical structures of both pyran copolymer and polyacrylic acid in my PhD thesis (Figure 2) [10].

For both pyran copolymer and polyacrylic acid, and synthetic anionic polymers in general [9], much more extensive pharmacologic studies will have to be carried out before any large-scale investigations in man, and, in particular, the long-term effects of the retention of these polyanionic substances in the reticuloendothelial system should be a matter of concern requiring further study [6].

## 3. Thiophosphate-Substituted Polyribonucleotides

**Figure 3:**
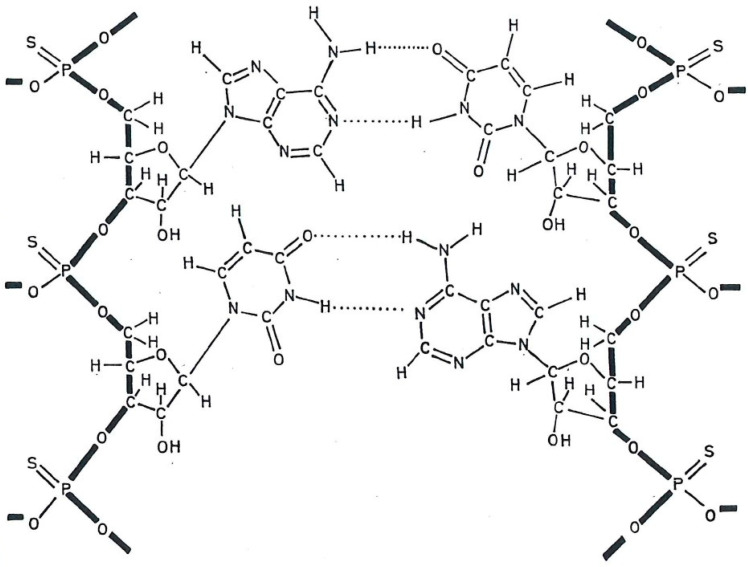
Molecular structure of thiophosphate analog of poly r(A-U): poly r($\bar{s}$A-$\bar{s}$U). Original work from E. De Clercq.

After a stable secondary, presumably multistranded, structure had been shown essential for the antiviral activity of polynucleotides [11], the thiophosphate-substituted polyribonucleotide poly r($\bar{s}$A-$\bar{s}$U) (Figure 3) or thiophosphate-substituted alternating copolymer of ribo adenylic–ribo uridylic acid, was found to be a more potent interferon inducer than its parent compound poly r(AU) [12]. Poly r($\bar{s}$A-$\bar{s}$U) was also much more resistant to degradation by pancreatic ribonuclease (as determined by residual antiviral activity) [12,13].

Incidentally, an active interferon inducer was isolated from *Hemophilus influenza* type B [14], although the chemical structure of this compound was not defined.

How the concept “interferon” grew after it had been first described by Isaacs and Lindenmann in 1957 [15] was addressed by De Clercq and Merigan in 1970 [16].

That the antiviral activity of polynucleotides could be increased by thermal activation was reported in 1970 [17]. However, in terms of interferon production in vitro and in vivo, substitution of thiophosphate for phosphate groups proved much more effective than thermal activation [17].

Interferon production is only one of the variety of host defense reactions initiated by interferon inducers, including toxicity, fever, increased phagocytosis, increased antibody formation, and hyperleukocytosis; these different host responses may be closely related phenomena [18]. Whether any of these interferon inducers may have clinical usefulness in therapy and/or prophylaxis [19] remains to be evaluated.

Where the role of interferon has been demonstrated is in the protective effect of poly(rI)∙poly(rC) against intranasal vesicular stomatitis virus (VSV) challenge in mice [20]. Interferon stimulated by double-stranded RNA should be further explored in the control of acute virus infections in man and domestic animals [21].

As originally shown for the alternating poly r(A-U), substitution of thiophosphate for phosphate in the alternating poly r(I-C) (ribo inosinic–ribo cytidylic acid) resulted in a significant increase in its ability to induce in vitro resistance to virus infection and interferon production in vitro and in vivo [13].

As to the thermal activation of the antiviral activity of synthetic double-stranded polyribonucleotides, it may have occurred through slippage of a branched helical structure to a longer unbranched structure [22]. Preincubation at 37 °C increased the resistance of poly r(A-U) to endonuclease (pancreatic ribonuclease) but did not change its sensitivity to exonucleases (venom phosphodiesterase) [22].

After Krueger and Mayer [23] and Mayer and Krueger [24] had reported that interferon could be induced in the mouse with a low-molecular-weight (mol. wt = 412) compound, tilorone hydrochloride, De Clercq and Merigan [25] confirmed this observation. The compound also protected young mice against an intranasal challenge with a lethal dose of VSV [25].

While the mechanism of interferon induction by synthetic double-stranded polyribonucleotides remains largely unresolved [26], no polynucleotide proved to be a more interferon inducer than poly(rI)∙poly(rC) [27,28]. Regretfully, poly(rI)∙poly(rC) was never subjected to any serious attempts to assess its usefulness in the prophylaxis and/or treatment of any viral infections in humans.

## 4. Host Defenses Against Viral Infections

**Figure 4:**
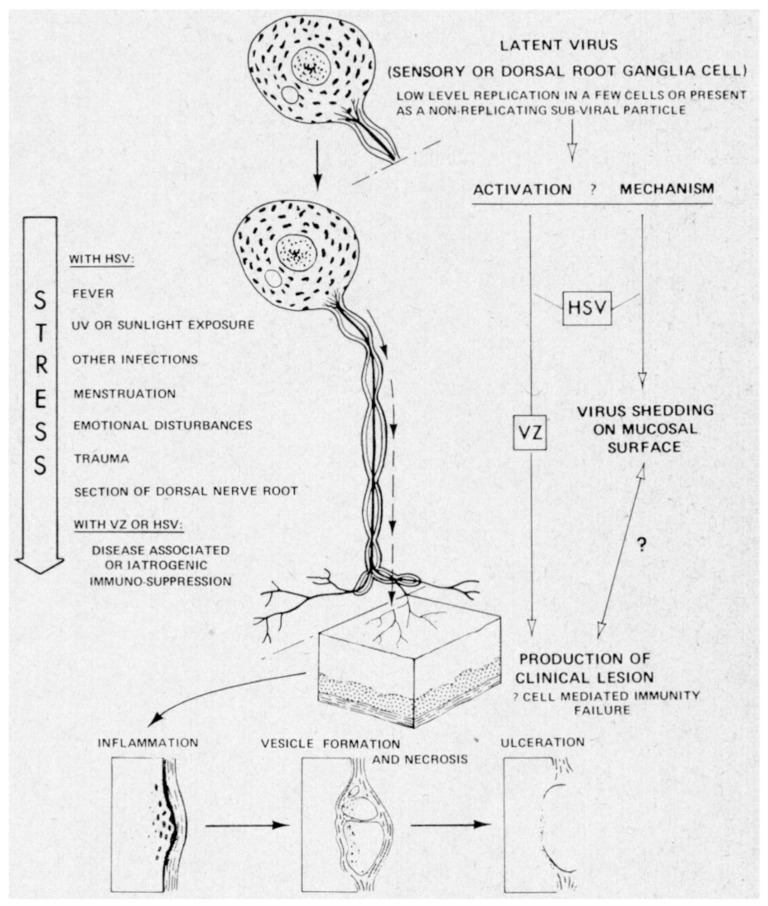
Mechanisms of reactivation of certain cutaneous herpes viruses. HSV represents herpes simplex virus, and VZ represents varicella-zoster virus. Reproduced with permission from T. C. Merigan, N Engl J Med; published by Massachusetts Medical Society, 1974 [31].

Topical (i.e., locally applied) interferon was attempted in the treatment of some respiratory virus (i.e., rhinovirus) infections [29] and human papillomavirus infections (i.e., condylomata acuminata) [30], but these initial studies were not followed up on. Instead, Merigan reflected on the increased understanding of the pathogenesis of human virus infections [31], as could be exemplified for herpes simplex virus (HSV) and varicella-zoster virus (VZV) (Figure 4).

That human interferon should be envisioned as a therapeutic agent was first proposed by Merigan in 1979 [32]. This viewpoint was again emphasized in 1983 [33], after Hirsch and his associates had shown that treatment with leukocyte α-interferon markedly reduced symptomatic cytomegalovirus (CMV) infection in kidney transplant recipients [34].

## 5. Human Leukocyte Interferon in the Treatment of VZV Infections

That T.C. Merigan was keenly interested in the treatment of VZV infections was evident when he and his colleagues reported that cytosine arabinoside (ara-C) did not offer a beneficial effect on the course of herpes zoster (VZV infection) in a controlled trial [35]. Preliminary studies were then undertaken with human leukocyte interferon in the treatment of herpes zoster [36]; the only adverse side effect of interferon being noted was fever of 38–40 °C after injection. Merigan then commented on the efficacy of adenine arabinoside (ara-A) in the treatment of herpes zoster [37], pertaining to a study conducted by Whitley et al. [38].

**Figure 5:**
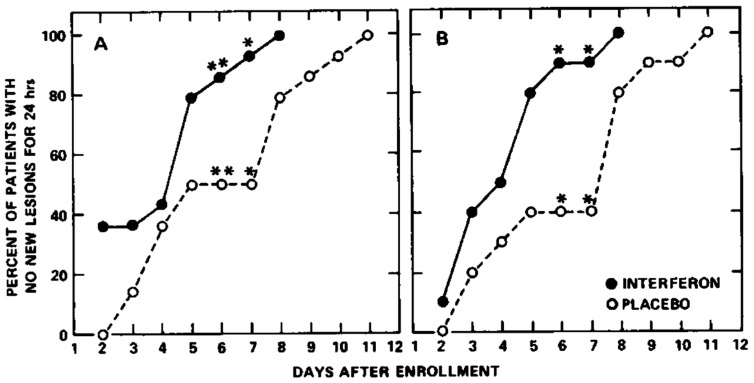
Percentage of patients with acute lymphocytic leukemia who had no new lesions for 24 h at each follow-up day. Reproduced with permission from A. M. Arvin, J. H. Kushner, S. Feldman, R. L. Baehner, D. Hammond and T. C. Merigan, N Engl J Med; published by Massachusetts Medical Society, 1982 [41].

In the treatment of varicella (VZV infection) in children with cancer, human leukocyte interferon diminished the number of complications from six out of nine to only two out of nine patients in a randomized double-blind placebo-controlled trial [39]. In three placebo-controlled, randomized double-blind trials involving 90 patients with cancer, human leukocyte interferon diminished the severity of post-herpetic neuralgia and limited cutaneous dissemination, visceral complications, and progression within the primary dermatome [40]. In the treatment of varicella in children with cancer, human leukocyte interferon effected a significant reduction in the number of days of new lesion formation [41] (Figure 5).

## 6. Interferon, ara-A, Acyclovir, and Ganciclovir in the Treatment of HSV and CMV Infections

In 1981, Chou et al. reported that viral cultures of mucocutaneous HSV lesions became negative in five heart-transplant patients given a 7-day course of intravenous acyclovir [42]. With the advent of various systemic antiviral agents, ara-A has been attempted in the treatment of CMV retinitis in immunosuppressed patients [43]. Also, human α-interferon has been tried in the treatment of CMV retinitis in AIDS patients [44]. With ganciclovir, very encouraging virological and clinical responses were obtained in the treatment of CMV infections in immunocompromised hosts [45]. Again, favorable results were obtained with ganciclovir in the outcome of serious CMV infections in heart and heart–lung transplant recipients [46].

**Figure 6:**
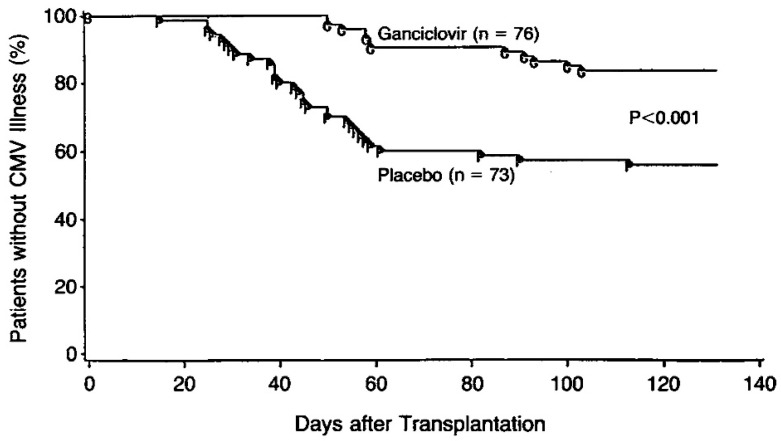
Incidence of CMV illness in the two study groups. Reproduced with permission from T. C. Merigan, D. G. Renlund, S. Keay, M. R. Bristow, V. Starnes, J. B. O’Connell et al., N Engl J Med; published by Massachusetts Medical Society, 1992 [48].

Merigan [47] warned that ganciclovir treatment of the AIDS patient with retinopathy should not be initiated until a definitive diagnosis is established. CMV, after all, is only one of several pathogens (i.e., Toxoplasma) that can produce lesions in the retina and choroid of the host. Yet, a controlled trial of ganciclovir proved efficacious in preventing CMV disease after heart transplantation [48] (Figure 6).

## 7. Interferon and ara-A in the Treatment of Chronic Hepatitis B Virus (HBV) Infection

Greenberg and associates reported that human leukocyte interferon was effective against HBV in patients with chronic active hepatitis [49].

**Figure 7:**
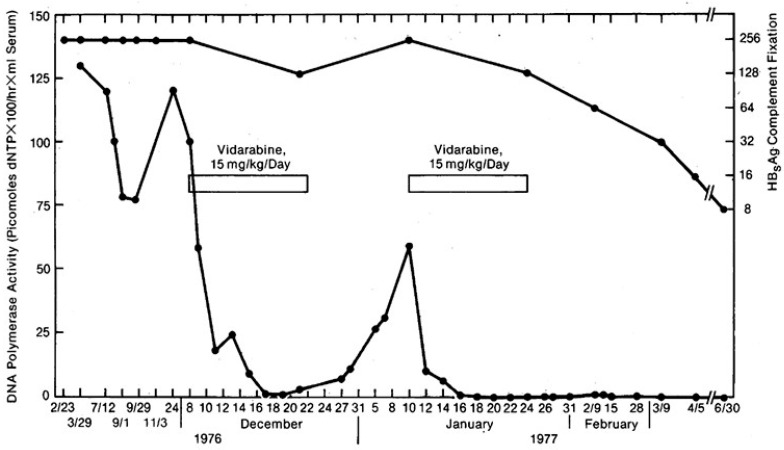
Effect of two courses of vidarabine on serum Dane particle DNA polymerase activity [(picomoles of deoxy nucleotides triphosphate (dNTP)] (lower line) and hepatitis B surface antigen (HB_s_AG) complement fixation titer (upper line) in a 22-year-old woman with insulin-dependent diabetes mellitus, known as HB_s_AG positive for 12 months, who never received treatment for chronic active hepatitis. Reproduced with permission from R. B. Pollard, J. L. Smith, A. Neal, P. B. Gregory, T. C. Merigan and W. S. Robinson, JAMA; published by American Medical Association (AMA), 1978 [50].

Then, Pollard and associates [50] showed that ara-A (vidarabine) administered to two patients with chronic active hepatitis B and persistently high levels of Dane particle DNA polymerase activity resulted in significant decreases in the levels of this Dane particle marker. The results obtained in one of these patients are depicted in Figure 7. A total of 12 of 32 patients with chronic hepatitis B virus infection lost HBeAg and DNA polymerase from their serum following treatment with interferon and/or ara-A [51], and three lost serum HBsAg as well.

In general, patients with chronic active hepatitis, those who are female and those with a history of recent steroid therapy responded to the antiviral therapy (interferon combined with ara-A) significantly better than did the other patients [52]; infectious virus could no longer be detected in patient serum after permanent responses to treatment with interferon and/or ara-A [53].

Although the clinical use of interferon and ara-A is compounded by toxic side effects [54], treatment of interferon combined with ara-A was still considered a promising therapy for chronic hepatitis B [55] (ara-A was, in the meantime, replaced by its 5′-phosphorylated derivative ara-AMP [56]). Also, recombinant forms of fibroblast β-interferon [57], as well as γ-interferon [58], have become available for the treatment of chronic HBV infections.

## 8. Treatment of HIV Infections

In 1984, in the early days after the description of AIDS, when the causative agent was tentatively defined as HTLV-III/LAV, Merigan oracled that “we will most probably need a combination of effective diagnostic tools, vaccines, and antiviral or immunoenhancing therapy (or both) to provide disease control for all who are at risk” [59]. Five years later, in 1989, he repeated this advice [60] about the efforts made in the treatment of the disease that in the meantime had been called human immunodeficiency virus (HIV) infection.

Recombinant soluble CD4 (rsCD4) was the first anti-HIV substance that Merigan was involved with [61]; it provided preliminary evidence of anti-HIV activity in vivo. Then, Merigan turned his attention to 2′,3′-dideoxycytidine (ddC) and found that both safety and activity could be maintained during long-term treatment with a low dose of ddC [62]. With zidovudine (AZT) and ddC, alternating and intermittent regimens were then attempted in the treatment of HIV infections [63]. After Merigan had been recommended the use of combinations of antiretroviral agents in the treatment of AIDS [64], he (and his colleagues) ascertained that alternating therapy with AZT and ddC reduced the toxicity associated with each drug while maintaining strong anti-HIV activity [65].

HIV-infected patients who developed the codon 74 mutation at codon 74 of the HIV reverse transcriptase gene after they had switched from AZT to 2′,3′-dideoxyinosine (didanosine, ddI) monotherapy had a greater serum virus burden than did patients without the codon 74 mutation [66]. Heterogeneity was observed among individual virologic responses to AZT and ddI combination therapy, and HIV resistance mechanisms during combination therapy appeared to be more complex than observed with monotherapy [67].

**Figure 8:**
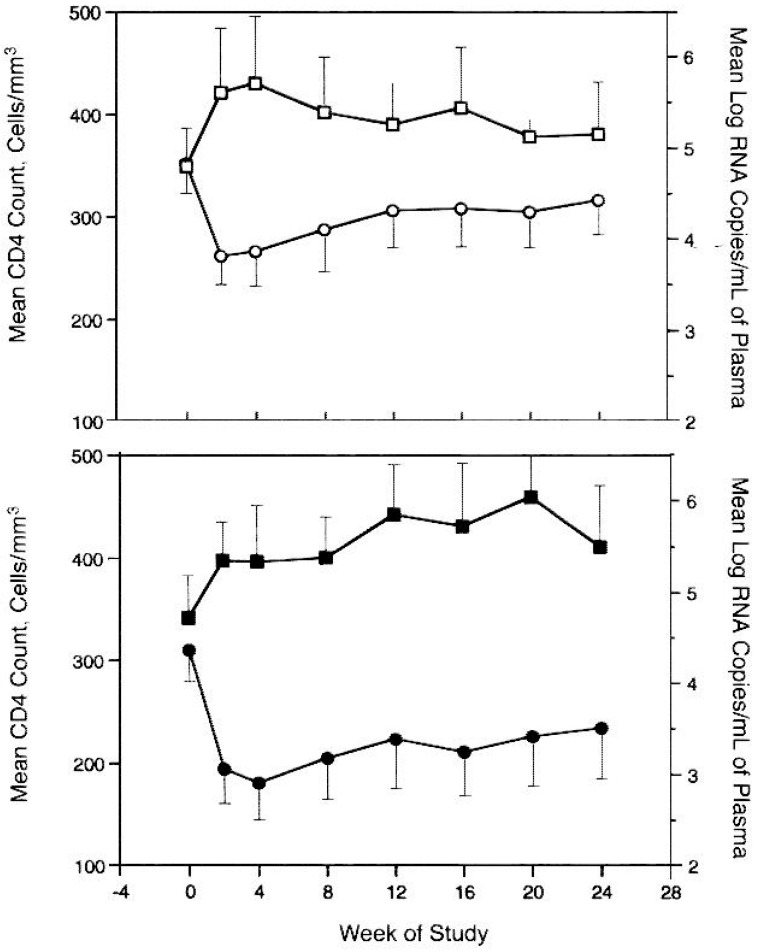
CD4+ T-cell counts and plasma human immunodeficiency virus (HIV) RNA levels over time in patients receiving saquinavir, 3600 mg/d or 7200 mg/d. Reproduced with permission from J. M. Schapiro, M. A. Winters, F. Stewart, B. Efron, J. Norris, M. J. Kozal et al., Ann Intern Med; published by American College of Physicians (ACP), 1996 [69].

After saquinavir had become available, its combination with ddC and AZT reduced HIV-1 replication and increased CD4^+^ cell counts more than did treatment with AZT and either ddC or saquinavir [68]. Saquinavir at a high dose (3600 or 7200 mg/day) caused a significant increase in CD4^+^ T-cell counts and a significant decrease in plasma HIV RNA levels over time [69] (Figure 8).

Top. Patients receiving 3600 mg/d. Open squares = CD4+ T-cell counts; open circles = plasma HIV-1 RNA levels. Bottom. Patients receiving 7200 mg/d. Solid squares = CD4+ T-cell counts; solid circles = plasma HIV-1 RNA levels. Bars indicate 95% Cls.

Treatment with AZT plus ddI, AZT plus ddC, or ddI alone proved superior to treatment with AZT alone in HIV-infected adults with CD4 T-cell counts from 200 to 500 per cubic millimeter [70].

Although drug resistance of HIV-1 is an obstacle to the long-term efficacy of antiretroviral therapy, some combinations of reverse transcriptase and protease mutations give the virus a selective advantage in the presence of various drug combinations [71].

The AIDS Clinical Trials Group 229 revealed a trend toward reduced mutations with three (AZT, ddC and saquinavir) versus two antiviral drugs (AZT and saquinavir) [72].

A 6-basepair insert between codons 69 and 70 of the reverse transcriptase gene conferred resistance to multiple nucleoside analogs [73].

When two sequential protease inhibitor regimens were used in saquinavir and NRTI (nucleoside reverse transcriptase inhibitor)-experienced persons, nelfinavir may have limited utility following saquinavir failure [74].

The T69D mutation in the HIV-1 reverse transcriptase (RT) gene has been associated with reduced susceptibility to ddC; however, several other mutations at codon 69 have been observed in antiretroviral drug-treated patients [75]. These variants included T69N, -S, -A, -G, -E, -I, and -K mutations that were present in patients treated with NRTIs but not in drug-naïve patients [75].

**Figure 9:**
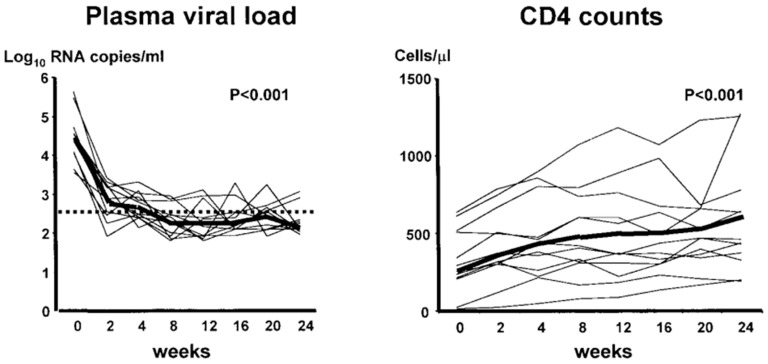
Time course of plasma viral load decrease, CD4^+^ cell count increase over 24 weeks in 13 patients receiving HAART. Reproduced with permission from C. M. Gray, J. Lawrence, E. A. Ranheim, M. Vierra, M. Zupancic, M. Winters, et al., AIDS Res Hum Retroviruses; published by Mary Ann Liebert, 2000 [77].

In patients treated with one protease inhibitor (PI) and two RT inhibitors, the sequential treatment with PIs selected for a relatively limited number of PI mutations that likely originated during early PI therapy [76]. Highly active antiretroviral therapy (HAART) resulted in plasma viral load decrease and CD4^+^ cell count increase over 24 weeks [77] (Figure 9).

As initial therapy for HIV-1 infection, M.S. Hirsch and colleagues [78] and T.C. Merigan and colleagues [79] judged the combination of zidovudine, lamivudine and efavirenz as superior to the other antiretroviral drug regimens. Antiretroviral drug regimens containing efavirenz and/or nelfinavir required further studies [80].

**Figure 10:**
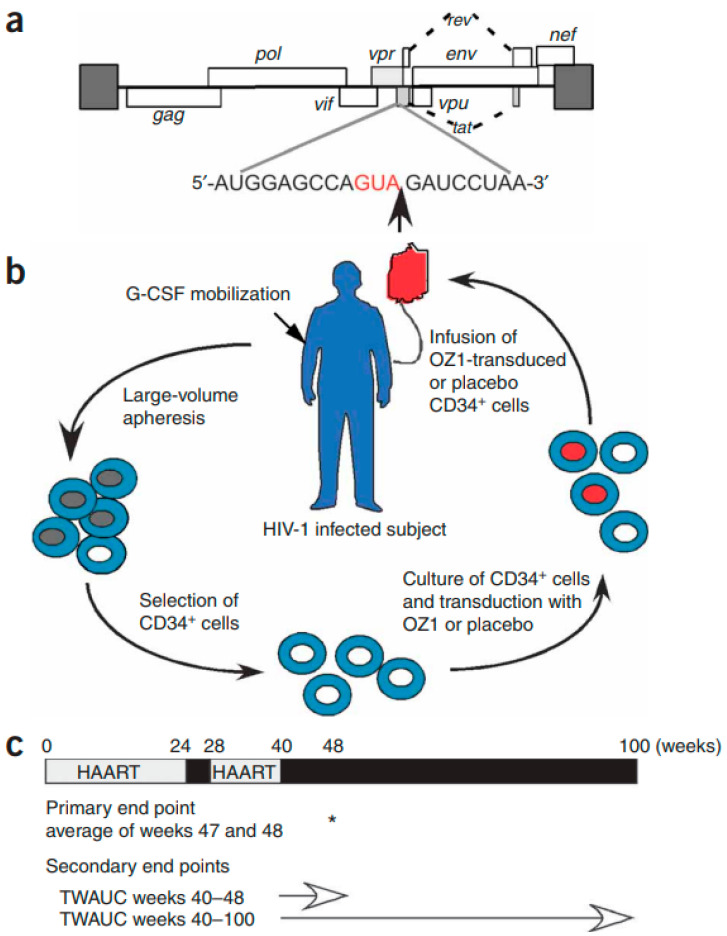
Ribozyme target site within HIV-1 genome, schematic of gene-modified cell manufacture and protocol design. (**a**) The HIV-1 genome is shown together with the target nucleotide sequence. The cleavage site is indicated by an arrow; cleavage is within an overlapping region of the *tat* and *vpr* genes. (**b**) This is a schematic of the process. HIV-1-positive individuals received G-CSF for stem cell mobilization, peripheral blood stem cells were collected by large volume apheresis on days 4 and 5, and CD34^+^ cells were selected, cultured and then transduced with OZ1 or placebo. On day 8, the cell product was washed and infused. (**c**) Participants continued HAART for 24 weeks after infusion before entering a 4-week treatment interruption (weeks 24–28), which was intended to apply selective pressure on any OZ1-containing cells. The analytical treatment interruption commenced after week 40 post-infusion. Assessments were conducted weekly during the analytical treatment interruption until week 48 and then monthly until either the resumption of HAART or week 100. Reproduced with permission from R. T. Mitsuyasu, T. C. Merigan, A. Carr, J. A. Zack, M. A. Winters, C. Workman et al., Nat Med; published by Nature Portfolio, 2009 [81].

Finally, Merigan and associates embarked on a randomized, double-blind, placebo-controlled, phase 2 cell-delivered gene transfer clinical trial in 74 HIV-infected adults who received a *tat-vpr*-specific anti-HIV ribozyme (OZ1) or placebo delivered in autologous CD34^+^ hematopoietic progenitor cells [81] (Figure 10). Throughout 100 weeks, CD4^+^ lymphocyte counts were higher in the OZ1 group than in the placebo group. This study indicated that cell-delivered gene transfer is safe and biologically active in individuals with HIV infection and could be developed as a conventional therapeutic product [81].

## Data Availability

No new data were created or analyzed in this study.
